# 2-Hy­droxy-3-meth­oxy­methyl-5-methyl­benzaldehyde

**DOI:** 10.1107/S1600536813002845

**Published:** 2013-01-31

**Authors:** B. Gunasekaran, A. Jayamani, N. Sengottuvelan, G. Chakkaravarthi

**Affiliations:** a Department of Physics & Nano Technology, SRM University, SRM Nagar, Kattankulathur, Kancheepuram Dist, Chennai 603 203 Tamil Nadu, India; b Department of Chemistry, DDE, Alagappa University, Karaikudi 630 003, India; c Department of Physics, CPCL Polytechnic College, Chennai 600 068, India

## Abstract

In the title mol­ecule, C_10_H_12_O_3_, all non-H atoms lie in a common plane (r.m.s deviation = 0.010 Å). The mol­ecular conformation is stabilized by an intra­molecular O—H⋯O hydrogen bond.

## Related literature

For the biological activity of methyl­benzene derivatives, see: Anbarasan *et al.* (2011 ▶); Chan & Daniels (2007 ▶). For related structures see: Wang *et al.* (2011 ▶); Kılıç *et al.* (2009 ▶); For graph-set notation of hydrogen bonds, see: Bernstein *et al.* (1995 ▶).
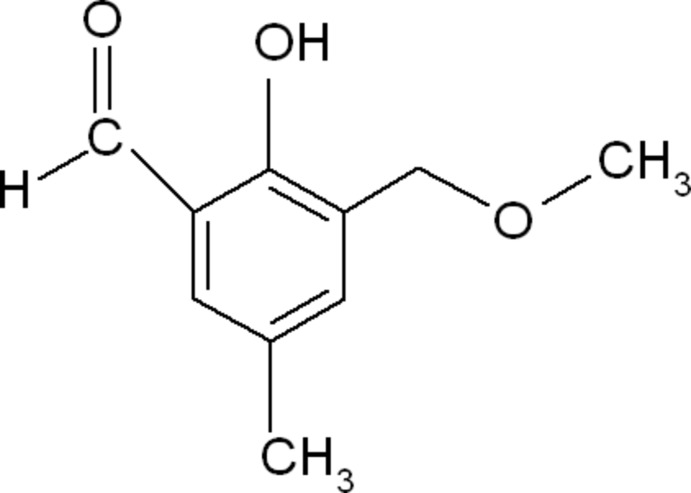


## Experimental

### 

#### Crystal data

C_10_H_12_O_3_
*M* *_r_* = 180.20
Monoclinic, 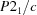

*a* = 13.899 (3) Å
*b* = 8.9184 (19) Å
*c* = 7.5043 (16) Å
β = 94.098 (6)°
*V* = 927.8 (3) Å^3^
*Z* = 4
Mo *K*α radiation
μ = 0.10 mm^−1^
*T* = 295 K
0.30 × 0.24 × 0.20 mm

#### Data collection

Bruker Kappa APEXII diffractometer
Absorption correction: multi-scan (*SADABS*; Sheldrick, 1996 ▶) *T*_min_ = 0.972, *T*_max_ = 0.981
10089 measured reflections
2329 independent reflections
1213 reflections with *I* > 2σ(*I*)
*R*_int_ = 0.050

#### Refinement

*R*[*F* ^2^ > 2σ(*F* ^2^)] = 0.054
*wR*(*F* ^2^) = 0.181
*S* = 1.03
2329 reflections
121 parameters
H-atom parameters constrained
Δρ_max_ = 0.24 e Å^−3^
Δρ_min_ = −0.22 e Å^−3^

### 

Data collection: *APEX2* (Bruker, 2004 ▶); cell refinement: *SAINT* (Bruker, 2004 ▶); data reduction: *SAINT* (Bruker, 2004 ▶); program(s) used to solve structure: *SHELXS97* (Sheldrick, 2008 ▶); program(s) used to refine structure: *SHELXL97* (Sheldrick, 2008 ▶); molecular graphics: *PLATON* (Spek, 2009 ▶); software used to prepare material for publication: *SHELXL97* (Sheldrick, 2008 ▶).

## Supplementary Material

Crystal structure: contains datablock(s) global, I. DOI: 10.1107/S1600536813002845/bt6886sup1.cif

Structure factors: contains datablock(s) I. DOI: 10.1107/S1600536813002845/bt6886Isup2.hkl

Supplementary material file. DOI: 10.1107/S1600536813002845/bt6886Isup3.cml

Additional supplementary materials:  crystallographic information; 3D view; checkCIF report

## Figures and Tables

**Table 1:** Hydrogen-bond geometry (Å, °)

*D*—H⋯*A*	*D*—H	H⋯*A*	*D*⋯*A*	*D*—H⋯*A*
O2—H2⋯O1	0.82	1.91	2.628 (3)	146

**Fig. 1.**
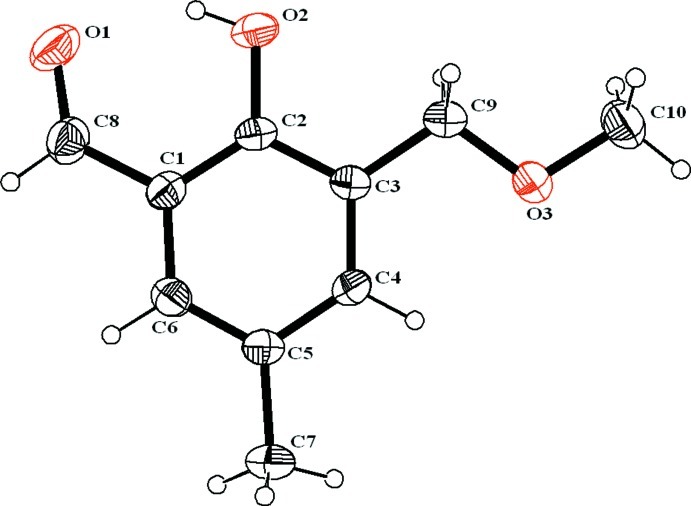
The molecular structure of (I), with atom labels and 30% probability displacement ellipsoids for non-H atoms.

## supplementary crystallographic information

### Comment

In recent days methylbenzene (toluene) and substituted methylbenzene have become very important on account of their wide range of applications in medicine and industry (Anbarasan *et al.*, 2011). For example 3-chloro-2-methylbenzene-1-sulfonylchloride shows the biological activity of hydroxysteriod dehydrogenase inhibitors (Chan & Daniels, 2007).

The geometric parameters of the compound (I), (Fig. 1) agree well with those of a reported similar structure (Wang *et al.*, 2011; Kılıç *et al.*, 2009) The molecular structure is stabilized by an intramolecular O-H..O interaction generating a six-membered ring S(6) graph-set motif (Bernstein *et al.*, 1995).

### Experimental

To a methanolic solution of 2-hydroxy-5-methyl-1,3-benzenedicarboxaldehyde (1g, 6mmol) decarborane (0.37, 3 mmol) was added slowly with constant stirring at room temperature in nitrogen atmosphere for 24 hours. A pale yellow solution formed after 24h and was concentrated to get the crude product. The crude product was washed well with methanol and dried in vacuum. The product was recrystallized in chloroform to get pale yellow coloured crystals suitable for single crystal XRD. yield 0.96g, 80% .

### Refinement

H atoms were positioned geometrically and refined using riding model with C-H = 0.93Å and *U*_iso_(H) = 1.2Ueq(C) for aromatic C-H, C-H = 0.97Å and *U*_iso_(H) = 1.2Ueq(C) for CH_2_, C-H = 0.96Å and *U*_iso_(H) = 1.5Ueq(C) for CH_3_ and O-H = 0.82Å and *U*_iso_(H) = 1.2Ueq(C) for OH.

### Crystal data

**Table:**

C_10_H_12_O_3_	*F*(000) = 384
*M**_r_* = 180.20	*D*_x_ = 1.290 Mg m^−^^3^
Monoclinic, *P*2_1_/*c*	Mo *K*α radiation, λ = 0.71073 Å
Hall symbol: -P 2ybc	Cell parameters from 3221 reflections
*a* = 13.899 (3) Å	θ = 2.7–24.8°
*b* = 8.9184 (19) Å	µ = 0.10 mm^−^^1^
*c* = 7.5043 (16) Å	*T* = 295 K
β = 94.098 (6)°	Block, yellow
*V* = 927.8 (3) Å^3^	0.30 × 0.24 × 0.20 mm
*Z* = 4	

### Data collection

**Table:**

Bruker Kappa APEXII diffractometer	2329 independent reflections
Radiation source: fine-focus sealed tube	1213 reflections with *I* > 2σ(*I*)
Graphite monochromator	*R*_int_ = 0.050
ω and φ scans	θ_max_ = 28.7°, θ_min_ = 2.7°
Absorption correction: multi-scan (*SADABS*; Sheldrick, 1996)	*h* = −18→18
*T*_min_ = 0.972, *T*_max_ = 0.981	*k* = −11→12
10089 measured reflections	*l* = −9→9

### Refinement

**Table:**

Refinement on *F*^2^	Primary atom site location: structure-invariant direct methods
Least-squares matrix: full	Secondary atom site location: difference Fourier map
*R*[*F*^2^ > 2σ(*F*^2^)] = 0.054	Hydrogen site location: inferred from neighbouring sites
*wR*(*F*^2^) = 0.181	H-atom parameters constrained
*S* = 1.03	*w* = 1/[σ^2^(*F*_o_^2^) + (0.0625*P*)^2^ + 0.4641*P*] where *P* = (*F*_o_^2^ + 2*F*_c_^2^)/3
2329 reflections	(Δ/σ)_max_ < 0.001
121 parameters	Δρ_max_ = 0.24 e Å^−^^3^
0 restraints	Δρ_min_ = −0.22 e Å^−^^3^

### Fractional atomic coordinates and isotropic or equivalent isotropic displacement parameters (Å^2^)

**Table:**

	*x*	*y*	*z*	*U*_iso_*/*U*_eq_	
C8	0.92734 (18)	1.0908 (3)	0.1482 (4)	0.0617 (7)	
H8	0.9842	1.1345	0.1959	0.074*	
C1	0.85176 (16)	1.1898 (2)	0.0792 (3)	0.0459 (6)	
C6	0.86550 (16)	1.3446 (3)	0.0868 (3)	0.0497 (6)	
H6	0.9241	1.3825	0.1349	0.060*	
C5	0.79476 (17)	1.4420 (2)	0.0250 (3)	0.0479 (6)	
C4	0.70806 (16)	1.3801 (2)	−0.0467 (3)	0.0457 (6)	
H4	0.6595	1.4449	−0.0906	0.055*	
C3	0.69089 (16)	1.2288 (2)	−0.0558 (3)	0.0426 (5)	
C2	0.76424 (16)	1.1324 (2)	0.0081 (3)	0.0438 (5)	
C9	0.59741 (17)	1.1631 (3)	−0.1290 (3)	0.0536 (6)	
H9A	0.6088	1.0965	−0.2275	0.064*	
H9B	0.5690	1.1049	−0.0370	0.064*	
C10	0.4451 (2)	1.2195 (3)	−0.2637 (4)	0.0741 (9)	
H10A	0.4569	1.1579	−0.3648	0.111*	
H10B	0.4030	1.3006	−0.3012	0.111*	
H10C	0.4155	1.1602	−0.1759	0.111*	
C7	0.8079 (2)	1.6091 (3)	0.0330 (4)	0.0687 (8)	
H7A	0.7929	1.6451	0.1483	0.103*	
H7B	0.7657	1.6556	−0.0576	0.103*	
H7C	0.8736	1.6336	0.0132	0.103*	
O1	0.92264 (14)	0.9535 (2)	0.1490 (3)	0.0772 (6)	
O2	0.74664 (13)	0.98329 (17)	0.0006 (3)	0.0618 (5)	
H2	0.7946	0.9376	0.0410	0.093*	
O3	0.53348 (12)	1.27783 (19)	−0.1888 (3)	0.0638 (5)	

### Atomic displacement parameters (Å^2^)

**Table:**

	*U*^11^	*U*^22^	*U*^33^	*U*^12^	*U*^13^	*U*^23^
C8	0.0507 (15)	0.0628 (17)	0.0718 (19)	0.0088 (12)	0.0061 (12)	0.0047 (13)
C1	0.0469 (13)	0.0412 (12)	0.0500 (14)	0.0053 (10)	0.0060 (10)	0.0027 (9)
C6	0.0463 (13)	0.0475 (14)	0.0549 (15)	−0.0047 (10)	0.0020 (10)	−0.0034 (11)
C5	0.0530 (14)	0.0374 (11)	0.0532 (14)	−0.0033 (10)	0.0039 (10)	−0.0027 (10)
C4	0.0475 (13)	0.0361 (11)	0.0535 (14)	0.0042 (9)	0.0028 (10)	0.0029 (10)
C3	0.0475 (12)	0.0357 (11)	0.0449 (13)	−0.0051 (9)	0.0059 (9)	0.0007 (9)
C2	0.0515 (13)	0.0319 (11)	0.0487 (13)	−0.0005 (9)	0.0086 (10)	0.0017 (9)
C9	0.0560 (14)	0.0406 (12)	0.0638 (16)	−0.0052 (11)	0.0027 (12)	0.0040 (11)
C10	0.0586 (17)	0.0758 (19)	0.085 (2)	−0.0147 (14)	−0.0139 (14)	0.0095 (16)
C7	0.0728 (18)	0.0374 (13)	0.095 (2)	−0.0071 (12)	−0.0003 (15)	−0.0078 (13)
O1	0.0704 (13)	0.0538 (12)	0.1080 (18)	0.0216 (9)	0.0109 (11)	0.0142 (11)
O2	0.0665 (12)	0.0333 (9)	0.0858 (14)	0.0009 (7)	0.0063 (10)	0.0045 (8)
O3	0.0500 (10)	0.0528 (10)	0.0858 (13)	−0.0075 (8)	−0.0136 (9)	0.0087 (9)

### Geometric parameters (Å, º)

**Table:**

C8—O1	1.227 (3)	C2—O2	1.352 (2)
C8—C1	1.440 (3)	C9—O3	1.407 (3)
C8—H8	0.9300	C9—H9A	0.9700
C1—C2	1.391 (3)	C9—H9B	0.9700
C1—C6	1.394 (3)	C10—O3	1.413 (3)
C6—C5	1.368 (3)	C10—H10A	0.9600
C6—H6	0.9300	C10—H10B	0.9600
C5—C4	1.398 (3)	C10—H10C	0.9600
C5—C7	1.503 (3)	C7—H7A	0.9600
C4—C3	1.372 (3)	C7—H7B	0.9600
C4—H4	0.9300	C7—H7C	0.9600
C3—C2	1.392 (3)	O2—H2	0.8200
C3—C9	1.494 (3)		
			
O1—C8—C1	125.2 (3)	O3—C9—C3	110.18 (18)
O1—C8—H8	117.4	O3—C9—H9A	109.6
C1—C8—H8	117.4	C3—C9—H9A	109.6
C2—C1—C6	119.6 (2)	O3—C9—H9B	109.6
C2—C1—C8	120.5 (2)	C3—C9—H9B	109.6
C6—C1—C8	119.9 (2)	H9A—C9—H9B	108.1
C5—C6—C1	121.5 (2)	O3—C10—H10A	109.5
C5—C6—H6	119.3	O3—C10—H10B	109.5
C1—C6—H6	119.3	H10A—C10—H10B	109.5
C6—C5—C4	117.3 (2)	O3—C10—H10C	109.5
C6—C5—C7	122.3 (2)	H10A—C10—H10C	109.5
C4—C5—C7	120.4 (2)	H10B—C10—H10C	109.5
C3—C4—C5	123.3 (2)	C5—C7—H7A	109.5
C3—C4—H4	118.3	C5—C7—H7B	109.5
C5—C4—H4	118.3	H7A—C7—H7B	109.5
C4—C3—C2	118.0 (2)	C5—C7—H7C	109.5
C4—C3—C9	123.2 (2)	H7A—C7—H7C	109.5
C2—C3—C9	118.74 (19)	H7B—C7—H7C	109.5
O2—C2—C1	121.9 (2)	C2—O2—H2	109.5
O2—C2—C3	117.8 (2)	C9—O3—C10	111.7 (2)
C1—C2—C3	120.25 (19)		
			
O1—C8—C1—C2	0.9 (4)	C8—C1—C2—O2	−0.1 (3)
O1—C8—C1—C6	179.6 (2)	C6—C1—C2—C3	0.2 (3)
C2—C1—C6—C5	−0.3 (3)	C8—C1—C2—C3	179.0 (2)
C8—C1—C6—C5	−179.0 (2)	C4—C3—C2—O2	179.3 (2)
C1—C6—C5—C4	−0.2 (3)	C9—C3—C2—O2	−0.1 (3)
C1—C6—C5—C7	179.6 (2)	C4—C3—C2—C1	0.2 (3)
C6—C5—C4—C3	0.7 (3)	C9—C3—C2—C1	−179.2 (2)
C7—C5—C4—C3	−179.1 (2)	C4—C3—C9—O3	1.4 (3)
C5—C4—C3—C2	−0.8 (3)	C2—C3—C9—O3	−179.2 (2)
C5—C4—C3—C9	178.7 (2)	C3—C9—O3—C10	178.3 (2)
C6—C1—C2—O2	−178.8 (2)		

### Hydrogen-bond geometry (Å, º)

**Table:**

*D*—H···*A*	*D*—H	H···*A*	*D*···*A*	*D*—H···*A*
O2—H2···O1	0.82	1.91	2.628 (3)	146
C4—H4···O3	0.93	2.38	2.736 (3)	103
